# Early detection of age related macular degeneration: current status

**DOI:** 10.1186/s40942-015-0022-7

**Published:** 2015-12-01

**Authors:** Roy Schwartz, Anat Loewenstein

**Affiliations:** Ophthalmology Division, The Sackler Faculty of Medicine, Tel-Aviv Sourasky Medical Center, Tel Aviv University, 6 Weizmann Street, Tel Aviv, 64239 Israel

**Keywords:** Age related macular degeneration (AMD), Choroidal neovascularization (CNV), Early detection, OCT, Amsler, Preferential hyperacuity perimetry (PHP)

## Abstract

Early diagnosis and treatment of choroidal neovascularization (CNV), a main cause of severe vision loss in age related macular degeneration (AMD), is crucial in order to preserve vision and the quality of life of patients. This review summarizes current literature on the subject of early detection of CNV, both in the clinic setting and mainly in the patient’s home. New technologies are evolving to allow for earlier detection and thus vision preservation in AMD patients.

## Introduction

Age-related macular degeneration (AMD) is the leading cause of blindness in persons older than 50 years in the United States and worldwide, accounting for 8.7 % of all legal blindness worldwide [1–4]. A main cause of severe vision loss due to the disease is the development of choroidal neovascularization (CNV), leading to the exudative or “wet” form of AMD. In patients presenting with CNV, treatment with intravitreal injections of anti-vascular endothelial growth factor (VEGF) agents may improve visual acuity (VA) by three lines or more in 30–40 % of patients and may prevent deterioration of visual acuity [5–10].

However, since the rise of different anti-VEGF agents, it has also become clear that early diagnosis and treatment is crucial for these outcomes, a fact which was demonstrated in many studies [5, 11–13]. For example, sub-analyses of the MARINA and ANCHOR studies revealed that the more early or immature a CNV lesion is, the better final VA outcome is expected following antiangiogenic treatment [6, 14]. Visual acuity at the time of initiation of anti-VEGF treatment was demonstrated to be the best predictor of VA at 1 and 2 years following treatment [5, 14]. It was shown that treatment of CNV within 1 month of detecting visual symptoms is more likely to result in visual acuity gain than treatment after this timeframe [13]. This may also apply to detection of advancing disease in the fellow eye. Recently published data from the Beaver Dam Eye Study showed that for eyes free of AMD in participants who were 50 years of age, the incidence of any AMD in that eye by 55 years of age was higher if AMD was present in the fellow eye (7 vs 2 %). A similar effect was seen in participants who were 70 years of age (21 vs 6 %) and in 90 year old participants (24 vs 10 %). Progression of the disease was more common as AMD severity progressed in the fellow eye [15].

It is therefore crucial that development of a new CNV be detected as early as possible, preferably before the development of a full-blown lesion which has already led to loss of letters, lines, and quality of life.

## Review

### Barriers to early detection of CNV

While it is clear that early detection of CNV in AMD patients is paramount for preservation of long term visual acuity, it is not always the reality outside of controlled studies. In a retrospective study of patients receiving the anti-VEGF agent ranibizumab, Rauch et al. found a mean time between initial symptoms and treatment of 59 ± 62 days [13]. In a different retrospective study by Canan et al., 73 % of patients had a symptom duration of 53.1 ± 14.2 days before commencing treatment [16].

During this lost time, the lesion keeps growing. CNV growth is typically accompanied by vision loss, with the majority of patients diagnosed with poor vision ranging from 20/63 [9, 17] in large studies to 20/138 in real world data [18]. Average lesion size of patients in large studies has an area of about 4000 microns and it was calculated that even the earliest enrolled patients in these studies had had neovascular AMD for 7.7 months prior to entry into the clinical trial [19].

The reasons for treatment delay are varied. First, progression of CNV can be rapid, with immature vessels reaching a maturation state within 10–14 days, [20] and patients may remain asymptomatic during this growth. Due to brain compensation mechanisms, the patient may not notice any change in vision in the early stages of the disease, [21] especially if the lesion develops outside the fovea. Second, public awareness to the disease is lacking. According to a large-scale survey published in 2003, only 3 of 10 adults in Western countries had any knowledge of AMD, and only 2 % knew that this disease is the leading cause of legal blindness [22]. This lack of knowledge, unfortunately, has not changed much since then, as evidenced by a recent study that found that 84 % of a sample of individuals with AMD in the United States were unaware of their disease [23].

### Current options for early detection of CNV

The current paradigm for early detection necessitates frequent monitoring, imaging [with optical coherence tomography (OCT) or fluorescein angiography (FA)] and clinical examination at the clinic. However, as was demonstrated above, the timeframe from appearance of symptoms to diagnosis and treatment is not ideal. Accordingly, several methods have been proposed for early detection of CNV, which may be unnecessarily delayed. These methods are described henceforth and are summarized in Table 1.

**Table 1:** Comparison of methods for early detection of CNV

Method	Central visual field evaluated	Description of method	Pros	Cons
OCT	NA	High resolution cross-sectional imaging of the retina	Sensitivity for detection of intra- and sub-retinal fluid in small quantities	Not yet portable, necessitating clinic visits
Amsler grid	20°	A chart consisting of a 10 cm square with a grid and a central spot for fixation	Readily available Convenient to use	Variable sensitivity
Near visual acuity	NA	Near vision chart	Readily available Convenient to use	Sensitivity and specificity were not studied
Preferential hyperacuity perimetry (PHP) home device	14°	Quantification of perceived distortions by the patient	Smaller decline in visual acuity compared to standard care	Cost
Shape-discrimination hyperacuity (SDH) smartphone application	NA	Shape discrimination tasks testing a patient’s ability to detect visual distortion	Convenient to use May differentiate between advanced and intermediate AMD	Large scale studies still underway at the time of this writing
Macular mapping test (MMT)	18°	Tests residual function while providing a quantitative score, allowing for monitoring of disease progression	May be useful to monitor progression of AMD	Not readily available No large scale studies
Noisefield perimetry	NA	Flickering dots are shown at high frequency. Patients report abnormalities in background noisefield	Relatively high sensitivity and specificity for detection of AMD, more at advanced stages	Not readily available No large scale studies

*CNV* choroidal neovascularization, *OCT* optical coherence tomography, *AMD* age related macular degeneration

### OCT

The development of OCT has changed the management of AMD patients. It provides detailed cross-sectional images of the retina, complementing data from funduscopic examination and FA, as was previously demonstrated by Coscas et al. [24]. Once diagnosed, OCT serves as an excellent tool for follow-up of CNV activity and its responsiveness to treatment.

Several studies appear in the literature on the subject of early detection of CNV using OCT. In a prospective, observational, nonrandomized pilot study by Padnick-Silver et al. [25]. Seventy-nine patients with neovascular AMD in one eye and non-neovascular AMD in the other were followed up every 3 months for 2 years. On each examination visual acuity and biomicroscopy were examined followed by Stratus OCT. If the OCT image raised suspicion, patients were reexamined 4–6 weeks later and/or FA was performed. Fifteen (19 %) developed CNV, of which 13 had disease progression identified on OCT before examination and/or FA showed changes.

De Sisternes et al. [26] developed a statistical model based on quantitative characteristics of drusen, including area, volume, height, and reflectivity on OCT that estimates the likelihood of conversion from early and intermediate AMD to advanced exudative AMD. Farsiu et al. [27] also used OCT biomarkers, in order to distinguish AMD from control eyes with a high accuracy. They delineated the retinal pigment epithelium (RPE) and RPE drusen complex (RPEDC, or axial distance from the apex of the drusen and RPE layer to Bruch’s membrane) and total retina (TR, or axial distance between the inner limiting and Bruch’s membrane) boundaries in OCT studies of 269 subjects with AMD from the Age-Related Eye Disease Study 2 (AREDS2) Ancillary SD-OCT study, and 115 control subjects without AMD. They showed that analyzing the topographic distribution of these disease indicators may serve as efficient biometrics to distinguish AMD from normal eyes.

Since the resolution of the Stratus, or time-domain OCT (TD-OCT) is lower than modern spectral domain OCT (SD-OCT) it is expected that newer machines would show even greater success in early detection of CNV. Indeed, in a review by the National Institute for Health Research (NHS), [28] seven studies reporting the accuracy of OCT in detecting active neovascular AMD were analyzed, five of which reported TD-OCT, [29–33] one reported SD-OCT, [34] and one reported both TD-OCT and SC-OCT [35]. For all OCT studies, the pooled sensitivity and specificity (95 % CI) was 85 % (72–93 %) and 48 % (30–67 %), respectively. For TD-OCT, the pooled sensitivity and specificity was 70 % (56–80 %) and 65 % (48–79 %), respectively. Reported sensitivities for the two SD-OCT studies were 94 and 90 %, and specificities were 27 and 47 %, suggesting that SD-OCT has higher sensitivity than TD-OCT but lower specificity.

OCT is currently the mainstay for CNV detection and AMD progression. Advantages of this method include the high sensitivity in detection of active disease, especially with newer, higher resolution, machines; the wide availability of OCT machines in retina clinics; and the relative speed and ease of use of the machine. Yet OCT has a disadvantage in the necessity of patients to attend the clinic in order to perform this exam. Even with the high sensitivity values reported, the time between appearance of CNV and its detection and treatment is suboptimal, as demonstrated previously.

Thus several ambulatory methods have been developed in the goal of detecting the lesion as early as possible, even at the patient’s home. These are described below.

### Amsler grid

**Fig. 1:**
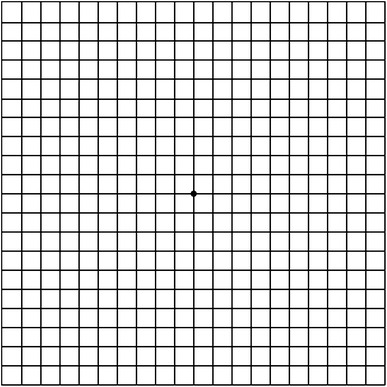
Amsler grid: first described in 1947 by Marc Amsler, this *chart* consists of a 10 cm square with a grid and a central spot for fixation

Amsler charts were first described in 1947 by Marc Amsler. [36] When used at a distance of 28–30 cm they evaluate the 20° of the visual field centered on fixation, [37] and they consist of a 10 × 10-cm square with a grid containing 400 single squares. The subdividing vertical and horizontal lines are 0.5 cm apart. Every single square represents an angle of 1°. A central spot for fixation is located in the center of the grid (Fig. 1). When the patient fixates on the central dot from a distance of approximately 30 cm, they are asked to report distortions, blurriness, or missing lines on the grid.

According to Amsler’s original work, [38–40] in patients with maculopathies subjective symptoms often precede objective signs, making the grid suitable for detection of macular disease at an early stage.

Amsler grids have the advantage of being readily available. Nowadays patients can make use of smartphone applications which show Amsler grids, making them even more accessible.

An increasing number of ophthalmologists, however, agree that the Amsler grid is not a sufficiently reliable tool with variable sensitivity for monitoring vision [41–43]. In a recent systematic review of 12 studies conducted by Faes et al. [44], it was found that the sensitivity of the test ranged from 0.34 to 1.0 and its specificity ranged from 0.85 to 1.0. Perhaps the most compelling reason for this test’s variable sensitivity is the remarkable capability of the brain to complete missing or out-of-line details, which could cause slight distortions to pass undetected [45]. Additionally, Amsler grids do not provide precise, quantifiable measures of visual field defects, and are therefore not useful as monitoring tools for disease progression.

### Near visual acuity

Research suggests that a reduction in reading rate is often noticeable among older patients prior to significant vision loss [46–49]. Indeed, one of the first signs or symptoms of visual loss in the early, non-NV stages of AMD, may be a reduction in reading rate, and near VA is a good predictor of reading rate [50]. As AMD progresses both near VA and reading rate reduce. It is thought that if near VA is a great deal worse than distance VA, a scotoma is impending near vision [48]. Therefore, some ophthalmologists provide their patients with a near-vision chart for home monitoring, in lieu of an Amsler chart or other CNV screening methods. This method, while having the advantage of being easily accessible, has not been proven in the literature.

### Preferential hyperacuity perimetry

Due to the shortcomings of the Amsler grid preferential hyperacuity perimetry (PHP) was developed [51]. Hyperacuity (also termed *vernier acuity*) is defined as the ability to perceive a difference in the relative spatial localization of 2 or more visual stimuli. It may detect miniscule changes in the relative localization of objects in space, within the central 14° of the visual field. RPE elevation and neurosensory retinal elevation, both possible occurrences in advanced AMD, causes a shift in the regular position of photoreceptors. It is hypothesized that such as shift causes an object to be perceived at a location different from its true location in space. This perceived shift, which may be the anatomical explanation for metamorphopsia, is recorded by PHP [52].

By presenting stimuli with artificial distortions of different amplitudes, a probabilistic estimation can be inferred: The collection of erroneous responses at the end of the test is used to identify, locate and quantify the size of perceived distortions. This quantification enables to distinguish between normal or quasi-normal distortions (e.g., as can occur because of large drusen) and larger distortions, more typical of CNV lesion [52]. The first generation of devices using PHP technology was designed for supervised use in a clinical setting. An algorithm was developed that distinguished between intermediate AMD and newly diagnosed CNV patients. Prospective multicenter studies done in 2003–2005 showed that this algorithm was able to discriminate between these two disease stages [52, 53]. However, there are limits to the use of PHP in the clinic setting, [54] including dependence on qualified clinical personnel, which restricts the frequency of visits per patient.

**Fig. 2:**
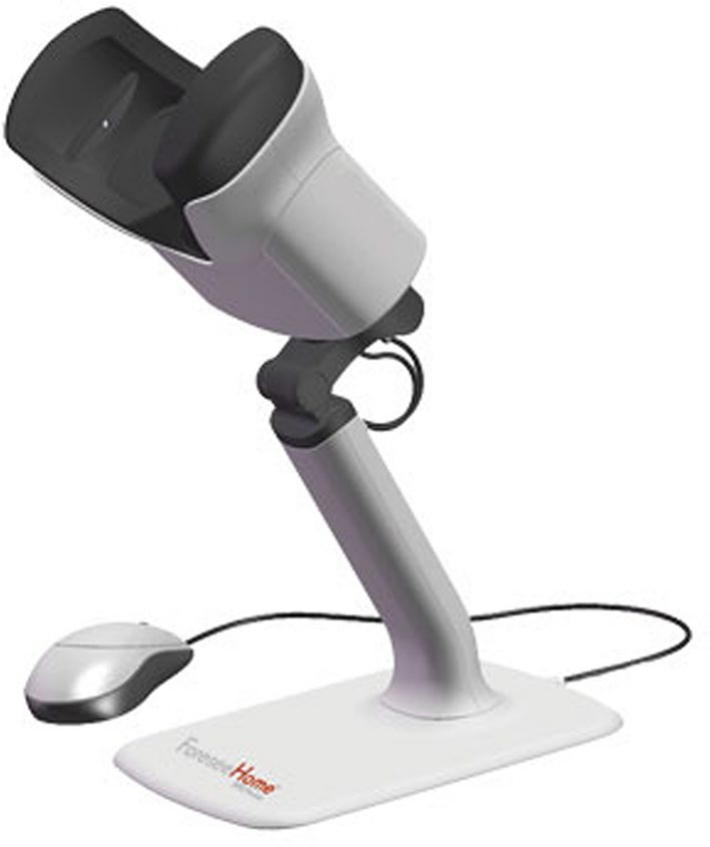
The ForeseeHome device: the device uses preferential hyperacuity perimetry for the early detection of CNV lesions

This has led to the development of a home device, after several modifications to the original device, including reduction in its physical size; enclosure of the screen viewer in a closed hood in order to control distance from the display, ambient light conditions, and occlusion of the non-tested eye; and addition of infrared sensors to ensure correct positioning of the head [55]. This device was evaluated in a phase III, unmasked, randomized clinical trial: the HOME (Home Monitoring of the Eye) study [56]. The study compared the use of the device, named ForeseeHome (Notal Vision, Israel) (Fig. 2), plus standard care, compared with standard care alone, for eyes at high risk of progression to CNV. Study participants at risk for developing CNV had either bilateral large drusen or large drusen in one eye with advanced AMD in the fellow eye. Forty-four AREDS2 clinical centers participated in the study. Standard care included investigator-specific instructions for self-monitoring at home (including aids such as an Amsler grid).

Of the 1970 participants in the study, 763 were randomized to device monitoring and 757 to standard care. They were followed for a mean of 1.4 years. Fifty-one patients in the device arm and 31 in the standard care arm progressed to CNV, with a smaller decline in VA from baseline to CNV detection in the device group [(median −4 (−11.0, −1.0) letters] compared with the standard care group [median −9 (−14.0, −4.0) letters] (p = 0.021). As a secondary visual acuity outcome, a higher percentage of eyes maintained 20/40 or better visual acuity at the time of CNV diagnosis and initiation of treatment in the device arm (87 %) compared with the standard care arm (62 %) (p = 0.014). Among participants who used the device at the recommended frequency, the proportion of eyes that maintained BCVA of 20/40 or better was 94 %.

These home-based devices have the advantage of allowing daily use without leaving the home, being simple to use, and sending information to a monitoring center. A possible disadvantage to the device is its high price, although reimbursement options are available.

### Shape-discrimination hyperacuity

A different form of hyperacuity measurement involves the discrimination of shapes. Using radial frequency (RF) patterns, Wilkinon et al. [57] demonstrated that humans have high sensitivity to sinusoidal deformation from circularity. The threshold for detecting radial deformation is a hyperacuity (<10 arcsec). Wang et al. [58] demonstrated that patients with early AMD had significant deficits in performing shape-discrimination tasks when compared with normal older subjects, without significant correlation with loss of VA. This dissociation between shape discrimination and VA suggested that this test may provide distinguishable information about the integrity of the photoreceptor mosaic in AMD.

A smartphone application for testing shape-discrimination hyperacuity (SDH) was recently introduced (MyVisionTrack, Vital Art and Science, USA). Wang et al. [59] evaluated 100 subjects, of which 37 had AMD, with the app. They found that measurements were higher in patients with advanced AMD than in those with intermediate AMD. The app has recently been approved by the FDA for use by prescription only. A pilot study was recently conducted on a remote monitoring system that utilizes the app [60]. The study was a single-arm, prospective, open-label, 16-week, multicenter study and was conducted at 24 centers in the US. It demonstrated that elderly patients with neovascular AMD were willing and able to comply with daily self-testing using the mobile device.

SDH shows promise as a monitoring tool as it necessitates only a smartphone, and may, as stated above, discover advanced stages of the disease on time. However, large scale studies regarding the sensitivity and specificity of the method are yet to be published. It is also possible that patients with advanced stages of the disease may have difficulty with recognizing patterns on a small phone screen.

### Macular mapping test

The macular mapping test (MMT) is designed mainly for quick assessment of residual vision in patients with maculopathies, but as it yields a quantitative score, it may be used as a tool for monitoring disease progression [61]. The MMT software displays a constant background pattern resembling a “wagon wheel” throughout the test, subtending the central 18° of the visual field. Eight spokes point toward the center of the display area, with a goal of aiding the patient with maintaining fixation at the center. Letters are briefly displayed on the screen and the patient is scored based on his ability to detect the letters.

Bartlett et al. [62] used this test to compare MMT scores between 31 healthy eyes of 31 participants, 17 age-related maculopathy (ARM), and 12 AMD affected eyes. They found a significant difference in the score between AMD affected eyes and controls (p < 0.001), showing that the device could be used as a tool for monitoring progression of the disease.

While MMT is a simple method for detection of disease progression, it is not readily available as a home-based machine, a smartphone app or computer based software. Large scale studies are also needed in order to prove its efficiency in the detection of CNV.

### Noisefield (Entoptic) perimetry

This technique, first introduced by Aulhorn in 1988, [63] uses random visual noise patterns. The test involves small black and white randomly flickering dots at a high frequency similar to television “static” or “noise”. The patient is asked to fixate on a central point and is asked to report abnormalities in the background noisefield. They may perceive a scotoma as a darker area, lighter area, or area of less motion.

Freemen et al. [64] tested this technique using scanning laser entoptic perimetry in 91 patients with AMD and 24 patients without AMD. They found an overall sensitivity of 82 % and specificity of 100 % for the detection of AMD. The sensitivity for early stages of the disease was greater than 70 % and increased to above 90 % for moderate to late stages. Koike [65] recently compared noisefield perimetry with Amsler grid for the differentiation of the dry and wet form of AMD. There was a trend towards lower sensitivity for noisefield perimetry (56 %) in comparison with Amsler grid (81 %) (p > 0.05). An opposite trend for higher specificity for noisefield perimetry (70 vs. 65 % for Amsler grid, p > 0.05) was shown. The combination of both tools was superior to either tool alone in terms of sensitivity, but not specificity, though without statistical significance.

Noisefield perimetry has the same disadvantages as MMT, not being a readily available tool for frequent home-monitoring, and lacking large scale trials to prove its usefulness.

### Future directions

The methods described above delineate the evolution of AMD monitoring, from simple methods such as the Amsler grid to sophisticated home-based machines and smartphone applications. Yet the field of home monitoring is continually evolving.

Such an evolution may involve OCT machines, currently only office-based. A new development in OCT machinery are swept-source OCT lasers, which are more readily portable than SD-OCT systems. This has led to the generation of the first prototype handheld OCT systems [66]. Another new concept is the field of binocular OCT, [67] using swept-source lasers to obtain simultaneous images from each eye in tandem, thereby removing the need for qualified personnel to acquire the image, as the patients align the optical axes of the instrument with the optical axes of their own eyes. Such machines have the potential of delivering both the sensitivity of OCT examination and the convenience and speed of testing at the home setting, and may revolutionize the field of home monitoring of CNV in the future.

A smartphone application was previously mentioned that uses SDH to monitor AMD patients. Indeed, the abundance of smartphones makes these tools a good option for always-available monitoring devices. Therefore, new methods are being developed that make use of these machines’ capabilities. A recent example is a pilot study by Winther and Frisén conducted on 28 patients with neovascular AMD of varying severity, using a MultiBit Test (MBT) [68]. It employs segmented digits defined by rarebits, or receptive field-size bright dots that are briefly presented against a dark background. Rarebit testing was devised with the aim of uncovering low degrees of neurovisual damage, where conventional tests often fail. Normal eyes are expected to see all rarebit probes, in central and peripheral vision. Conversely, eyes with losses of receptive fields will miss some probes.

In the study, subjects used a smartphone/tablet application. The application generates rarebits, and their number varies in a cyclic fashion, in preset steps. This test presents no fixation demands. Patients were monitored for an average of 30 weeks and results were compared with the clinical status recorded on clinical examinations. Plots of MBT results showed gradual improvement after successful antineovascular treatment, while recurrences were seen as gradual deteriorations of results.

## Conclusions

The unmet need for early detection of the wet form of AMD has led to the development of several promising technologies for the detection of CNV. These techniques continue to develop and will allow the retina specialist to improve patient care. Large-scale studies such as the HOME Study validate that early detection of CNV can surpass conventional, standard care methods with greater sensitivity for identifying early lesions. Advances in applications for home use devices to detect CNV show promise for improving disease outcomes in patients with AMD.
